# Bioinformatic prediction, deep sequencing of microRNAs and expression analysis during phenotypic plasticity in the pea aphid, *Acyrthosiphon pisum*

**DOI:** 10.1186/1471-2164-11-281

**Published:** 2010-05-05

**Authors:** Fabrice Legeai, Guillaume Rizk, Thomas Walsh, Owain Edwards, Karl Gordon, Dominique Lavenier, Nathalie Leterme, Agnès Méreau, Jacques Nicolas, Denis Tagu, Stéphanie Jaubert-Possamai

**Affiliations:** 1 INRA, UMR1099 BiO3P, Domaine de la Motte, F-35653 Le Rheu, France; 2 INRIA Centre Rennes - Bretagne Atlantique, GenOuest, Campus de Beaulieu, 35042 Rennes, France; 3 Université de Rennes I/IRISA Campus de Beaulieu, 35042 Rennes, France; 4 CSIRO Entomology, GPO Box 1700, Canberra ACT 2601, Australia; 5 CSIRO Entomology, Centre for Environment and Life Sciences (CELS), Floreat Park, WA, 6014, Australia; 6 ENS Cachan/IRISA Campus de Beaulieu, 35042 Rennes, France; 7 CNRS, Univ Rennes 1, UMR 6061, IFR 140, Rennes, France

## Abstract

**Background:**

Post-transcriptional regulation in eukaryotes can be operated through microRNA (miRNAs) mediated gene silencing. MiRNAs are small (18-25 nucleotides) non-coding RNAs that play crucial role in regulation of gene expression in eukaryotes. In insects, miRNAs have been shown to be involved in multiple mechanisms such as embryonic development, tissue differentiation, metamorphosis or circadian rhythm. Insect miRNAs have been identified in different species belonging to five orders: Coleoptera, Diptera, Hymenoptera, Lepidoptera and Orthoptera.

**Results:**

We developed high throughput Solexa sequencing and bioinformatic analyses of the genome of the pea aphid *Acyrthosiphon pisum *in order to identify the first miRNAs from a hemipteran insect. By combining these methods we identified 149 miRNAs including 55 conserved and 94 new miRNAs. Moreover, we investigated the regulation of these miRNAs in different alternative morphs of the pea aphid by analysing the expression of miRNAs across the switch of reproduction mode. Pea aphid microRNA sequences have been posted to miRBase: http://microrna.sanger.ac.uk/sequences/

**Conclusions:**

Our study has identified candidates as putative regulators involved in reproductive polyphenism in aphids and opens new avenues for further functional analyses.

## Background

MicroRNAs (miRNAs) are small (18-24 nucleotides) non-coding RNAs (ncRNAs) that regulate gene expression in eukaryotes. Numerous miRNA genes have been found in animal genomes. They are located either within intronic sequences of mRNA-coding genes, or in intergenic regions. Many miRNAs are highly conserved throughout evolution (reviewed in [1]). However, there are also taxa-specific miRNAs [2-5]. One such miRNA is iab-4 which is only described in insect species and is involved in wing formation [6]. The description of insect miRNAs in miRBase [7] remains largely restricted to Diptera (*Drosophila melanogaster, D. pseudoobscura*, *Anopheles gambiae*), Hymenoptera (*Apis mellifera, Nasonia vitripennis*), Coleoptera (*Tribolium castaneum*), Orthoptera (*Locusta migratoria*) and Lepidoptera (*Bombyx morii*), which all diverged about 280 million-years ago [8]. The insect species with the greatest number of miRNAs (147) represented in miRBase is *D. melanogaster*, which until recently was also the only insect for which deep sequencing results have been combined with a thorough bioinformatics analysis [9]. This number has recently been surpassed in a study on the silkworm, *B. mori *[10] and the locust *Locusta migratoria *[11]. Functional analyses of insect miRNAs has been mainly restricted to *D. melanogaster *[9,12] where they have been shown to affect multiple biological processes such as embryo development and tissue differentiation, cell proliferation or morphological changes. Until now, no miRNAs have been described from the Hemiptera, a group of insects that includes many of the world's most damaging insect pests such as aphids, whiteflies, and scales.

MiRNAs are generated from genome-encoded precursors that form hairpin structures with imperfect base-paired segments. MiRNAs processing occurs in several steps (reviewed in [13,14]). The primary transcripts (pri-miRNA) are essentially synthesised by RNA polymerase II and cleaved in the nucleus by the RNAse III Drosha. The resulting 70 nucleotide-hairpin pre-miRNA is exported in the cytoplasm and the final miRNA maturation step is mediated by the RNAse III Dicer that produces 18-24 nucleotide long miRNA duplexes. One strand of this duplex is incorporated into the RNA-induced silencing complex (miRISC) and guides this miRISC to degrade, destabilize or translationally inhibit the mRNA targets (reviewed in [9]). Post-transcriptional regulation of gene expression by miRNAs is highly complex since a single miRNA can regulate hundred of genes, and a single gene may be regulated by multiple miRNAs.

The newly assembled genome of the pea aphid *Acyrthosiphon pisum *recently released by the International Aphid Genomics Consortium [15] has enabled the identification of miRNAs in a hemipteran insect for the first time. Aphids are herbivorous insects characterised by the unique ability to feed exclusively on phloem sap. The serious damage caused by aphids is partly due to their amazing ability to adapt to environmental variations [16]. This adaptive capacity is largely explained by their phenotypic plasticity that allows the production of distinct phenotypic morphs in response to environmental changes. This is illustrated by the switch of reproductive mode in response to seasonal changes: parthenogenetic females are produced in spring and summer whereas sexual females and males are produced in autumn [reviewed in [17]]. The pea aphid also shows a unique duplication of the miRNA processing machinery suggesting that miRNAs play a significant role in the life cycle of these insects [18]. Here, we present a list of 149 *A. pisum *miRNAs identified by combining *in vivo *and bioinformatic approaches. Moreover, the regulation of these miRNAs in phenotypic plasticity has been investigated and miRNAs known to be regulated by insect endocrine pathways were identified as differentially expressed in different morphs involved in sexual and asexual reproduction.

## Results and discussion

### Identification of *A. pisum *miRNAs

The recently sequenced and assembled *A. pisum *genome [15] was used to identify miRNAs in the pea aphid using three complementary strategies. First, miRNAs listed in miRBase were used to identify pea aphid miRNAs by sequence homology. Second, small RNAs extracted from parthenogenetic females of the pea aphid were sequenced by deep sequencing technology and analysed using the miRDeep program [19]. Finally, the pea aphid genome was screened for putative miRNA precursors using a new algorithm, GR4500.

In the first approach we blasted 1275 insect miRNAs (*D. melanogaster *and 11 other *Drosophila *species, *A. gambiae, B. mori, T. castaneum*, *A. melifera*, *Locusta migratoria*) deposited in miRBase release 14 against the *A. pisum *genome. The 200 nucleotide-long genomic sequences surrounding each hit were investigated for their ability to fold into typical miRNA precursor hairpins. In total 43 different putative aphid miRNAs showed both sequence homology and a potential hairpin structure (Additional file 1). These 43 *A. pisum *miRNAs candidates corresponded to 44 precursors. The expression of 33 of them was verified by RT-PCR (data not shown).

In the second approach we undertook deep sequencing of small RNAs from a mixed generation sample of parthenogenetic female pea aphids. Approximately 3 million sequences were generated, corresponding to approximately 850,000 unique sequences. MiRNAs are known to show obvious size preference and tend to be between 18 and 24 nucleotides in length. Analysis of the length distribution of the pea aphid mature miRNAs showed a peak at 22nt as previously reported in *D. melanogaster*. Moreover, similar to other organisms, many of the predicted pea aphid miRNA mature sequences (59%) start with a uridine residue at their 5'end [20-22].

**Figure 1:**
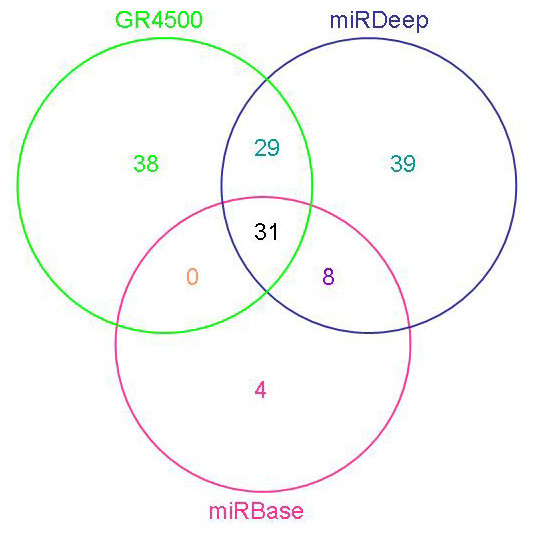
**Venn diagram showing the distribution of predicted miRNAs in the pea aphid from three different methods: homology to miRBase, miRDeep or GR5400**.

These sequences were mapped on the pea aphid genome and analysed by miRDeep, which uses a probabilistic model of miRNA synthesis by the Dicer protein to score compatibility and frequency of small RNA sequences with the secondary structure of the pre-miRNA genomic precursor [19]. 127 sequences (0.16% of the 850,000 initial sequences) were identified as potential miRNAs corresponding to 107 unique *A. pisum *miRNAs (Additional file 1). Of these, 25 corresponded to miRNAs already identified by the homology search in miRBase (Fig 1). For the 82 remaining sequences, we checked for orthologs in other insect species, by searching for sequences homologous to the predicted precursors among the other available insect genomes. We also searched for orthologs by comparing *A. pisum *predicted precursors to the sequences of mature miRNAs listed in miRBase. This analysis allowed the identification of 11 precursors, identified by miRDeep and GR4500 that matched to miRNA listed in miRBase. However, the predictions of miRDeep and GR4500 for these 11 miRNAs appeared to be the complementary strand mir* of conserved miRNAs (mir 10*-mirX3; mir14*-mirX6; mir137*-mirX1; mir219*-mirX17; mir281*-mirX7; mir965*-mirX21, mir993*-mirX42, mir316*-mirX46, mir276*-mir276a; mir9c2*-mirX16, mir13b*-mirX53). Among these 11 conserved miRNAs, two (mir-9c2 and mir-13b) were missed by our first approach based on homology search because of the high stringency of our blast analysis. One of the miRDeep prediction parameters stated that the larger number of short-reads mapping to the stem corresponds to the mature miRNA and that the opposite strand corresponds to the mir* sequence. This is based on the assumption that mature miRNAs are more abundant that mir* in living cells. However, stronger expression of mir* compared to the mature miRNA has been reported in various organisms [11,23-25] and more and more evidences support a function for mir* as regulatory RNAs [25,26]. This could also be also the case for some of the predicted miRDeep miRNAs in the pea aphid.

MiRDeep is a selective program for identifying miRNAs from deep sequencing data that minimises the false positive rate. It selects reads that align exactly to the genome, at less than 5 different locations, and selects putative precursors of miRNA that match a high number of reads on the hairpin structure where the mature miRNA is supposed to be. Using a pool of several 454 sequencing runs, Friedländer et al. [19] showed that miRDeep was able to retrieve 89% of the known miRNA from *Caenorhabditis elegans*, and recovered 73% of the known miRNA incorporated in a set of 10^6 ^Solexa sequences from a HeLa cell small RNA sample sequenced by Solexa technology. In addition to miRDeep, in order to increase the number of predicted pea aphid miRNAs, we implemented a complementary approach, called GR4500. This algorithm first screened the pea aphid genome for miRNA putative precursors (hairpin structure, see methods) using a classifier trained with a set of 30 validated pea aphid miRNAs. GR4500 then compared the selected hairpins to the Solexa reads for biological validation and for identifying the mature sequence. The classifier selected 4402 candidates from 2.5 million genomic hairpins of more than 63 nucleotides. The comparison of these candidates with the 850,000 sequences of small RNAs previously obtained by deep sequencing confirmed the expression of 116 of them. These 116 precursors corresponded to 98 different mature miRNAs (Additional file 1). GR4500 identified 38 new mature miRNAs not found in miRBase or identified by miRDeep from 45 genomic precursors. None of these 38 miRNAs have orthologues in other organisms.

Altogether, the combination of homology search, miRDeep analysis of deep sequencing and GR4500 scan of the genome allowed the identification of 149 mature miRNAs candidates in the pea aphid (Fig 1). These 149 mature miRNAs candidates correspond to 176 precursors of miRNAs (pre-miRNAs) (Additional file 1). This is similar to the number of annotated miRNAs on miRBase for *D. melanogaster *(157 miRNAs). For all these pea aphid miRNA candidates, we have evidence of their expression since mature sequences have been detected either by RT-PCR and/or deep sequencing. It is admitted that microRNAs identified by deep sequencing, have to be supported by multiple reads and/or by the existence of its complementary strand mir* (Meyers *et al*. 2008). Applying this rule to the pea aphid predicted miRNA by miRDeep and GR4500 lead to the subdivision between 98 *A. pisum *miRNAs (≥ 5 reads and/or mir* detected) and 51 miRNA candidates (< 5 reads without mir*) (Additional file 2). These miRNA candidates still require confirmation that will be provided by future extensive deep sequencing, such as in other morphs than parthenogenetic individuals used for this study.

### Genomic organisation of miRNA precursors

As expected, the majority of miRNA precursors (86%) were located in intergenic regions (97 precursors) or introns (55 precursors) With the exception of two miRNAs located in unassembled reads (raw sequences from the genome of the pea aphid not assembled in the first release of the genome, see IAGC et al. 2009) (Additional file 1), the remainders of the miRNA precursors were located in regions annotated as exons from protein coding genes (12.5%, 22). However, only two of these correspond to predicted genes whose annotation is supported with biological evidence (ESTs), encoding a heterogeneous nuclear ribonucleoprotein K and a vesicular mannose-binding lectin. Exonic miRNAs have been previously described in mammals [27,28]. However, with a few rare exceptions, exons that encode miRNAs do not code for proteins and are "mRNA-like non coding RNAs". The remaining 20 exonic miRNAs of the pea aphid are found in predicted genes whose annotation is not yet supported by biological evidence. Moreover, among the 20 miRNAs identified within exon, 15 were well supported either by abundant Solexa reads and/or the existence of mir*. This could indicate false prediction in the protein coding gene set, or that these predicted genes correspond to mRNA-like non coding RNAs.

We investigated the physical distribution of the 176 precursors of miRNAs along the different assembled genomic scaffolds of the pea aphid genome. 71.6% (126) of miRNA precursors were identified as singletons (1 miRNA locus per scaffold) while 28.4% (50) of miRNAs precursors were distributed in 17 clusters composed of up to 6 miRNAs per cluster (Additional file 1). Among the 17 pea aphid miRNAs clusters, 3 clusters are composed of the same identical mature miRNAs present as multicopy (Ap-mir-X40, Ap-mir-971, Ap-mir-X5) with slight differences within precursor sequences, suggesting a very recent duplication of these miRNAs. We identified 4 clusters composed of closely related miRNAs (e.g. Ap-mir92a and Ap-mir-92b) with slight differences in their precursor or mature sequences, and 10 clusters composed of different mature miRNAs, suggesting more ancient duplication events. The length of these clusters varies from 160 nucleotides (in that case, the miRNA precursors overlap) to 30 kb.

Two aphid miRNA clusters are unusually long: cluster EQ127026-cl1 (Ap-mir-277/Ap-mir-317/Ap-mir-34) at 15 kb and cluster EQ127560-cl1 (Ap-mir-263b/Ap-mir-228) at 30. This EQ127026-cl1 cluster is also conserved in *D. melanogaster *and *A. mellifera*. Moreover, long length miRNA clusters up to 62 kb have been identified in the mouse genome [29]. Cluster EQ119865-cl1 (Ap-mir-2a-1a/Ap-mir-2a-1b/Ap-mir-2b/Ap-mir-13a/Ap-mir-13b/Ap-mir-71), and cluster EQ127026-cl1 also show similar organisation between the honey bee and the pea aphid genomes. However, some clusters conserved in *D. melanogaster *and honey bee have diverged in aphids. The duplication of Api-mir-307 observed in the pea aphid genome has not been reported in other insect genomes. The cluster including let-7, mir-100 and mir-125 conserved in *D. melanogaster *and in *A. mellifera *is incomplete in *A. pisum*, since Api-mir-125 was not found. Sequence analysis of the scaffold EQ112277-cl1 containing Ap-mir-100 and Ap-let-7 showed a sequence partially homologous to mir-125 (18 bases identical on 22). However, the genomic sequence surrounding this putative mir-125 sequence did not show any hairpin structure. These variations in clusters organisation could indicate an adaptation for each insect order of their miRNA gene contents possibly related to life history traits.

### Functional annotation of pea aphid miRNAs

**Figure 2:**
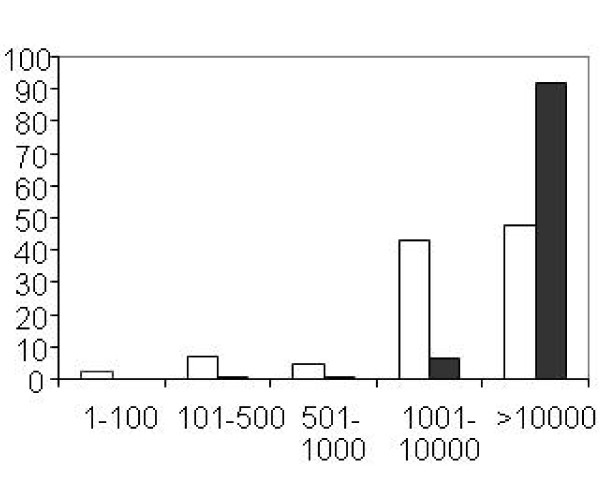
**Frequency of sequence reads among the pea aphid miRNAs**. Those miRNAs that are also found in other species show higher read frequencies than the pea aphid specific miRNAs. For the two batches of miRNAs (in black, miRNAs found in other species and in white miRNAs found only in *A. pisum*), the number of reads have been distributed between 5 classes: miRNAs represented by 1-100 reads, 101-500 reads, 501-1000 reads, 1001-10000 reads or >10000 reads. The figure shows for each batch the percentage of miRNAs in each class.

The 149 mature aphid miRNAs were compared to other identified miRNAs by using the homology based SEARCH program available at miRBase. Only 55 (37%) of the 149 aphid mature miRNAs showed significant homology with known miRNAs. The other 94 miRNAs showed no significant homology to previously described miRNAs and were designated Ap-mirX. As not all putative miRNAs from insect species are in miRBase, we searched for highly similar nucleotide sequences within whole insect genomes: *D. melanogaster*, *A. mellifera*, *T. castaneum*, *A. gambiae *and *N. vitripennis*. From the 94 putative new miRNAs identified in the pea aphid genome, 29 similar sequences were found in other insect genomes. The remaining 65 miRNAs are detected only in the pea aphid (Additional file 1); in parallel, most (over 100) miRNAs identified in *D. melanogaster *were not found in the pea aphid. This high proportion of non-conserved miRNAs among species is not surprising given the recent results obtained from different deep sequencing analyses of small RNAs performed on several species: the correlation between sequence conservation and high expression level often observed for many miRNAs before the development of deep sequencing erroneously led to the general conclusion that miRNAs are highly conserved. However, the identification of low expressed miRNAs allowed by deep sequencing suggested to Glazov and collaborators [24] that the only the most expressed miRNAs corresponded to conserved miRNAs. In the case of the pea aphid, we observed that on average, the new miRNAs that have no homolog in other organisms are less represented in the Solexa short reads than the conserved miRNAs (Fig 2; Wilcoxon test t p-value 2.161e^-11^). This suggests that these new miRNAs are less expressed and confirms the hypothesis of Glazov et al. [24].

### Expression of miRNAs in alternative pea aphid morphs

Aphids have a complicated life cycle characterized by a phase of viviparous clonal reproduction (parthenogenesis) that alternates with a phase of sexual reproduction. This switch is triggered by a sensing of the decrease of the day length in autumn (reviewed in [17]). The different alternative morphs produced are: parthenogenetic females named virginoparae producing viviparous parthenogenetic females, oviparae sexual females, sexual males, and parthenogenetic females named sexuparae producing sexual males and females. The mechanisms by which aphids detect and respond to differences in day length are not known. MiRNAs have been implicated in modulating circadian rhythm responses in insects [30]. On this basis, it is reasonable to hypothesize that miRNAs may also contribute to the regulation of aphid polyphenism.

**Figure 3:**
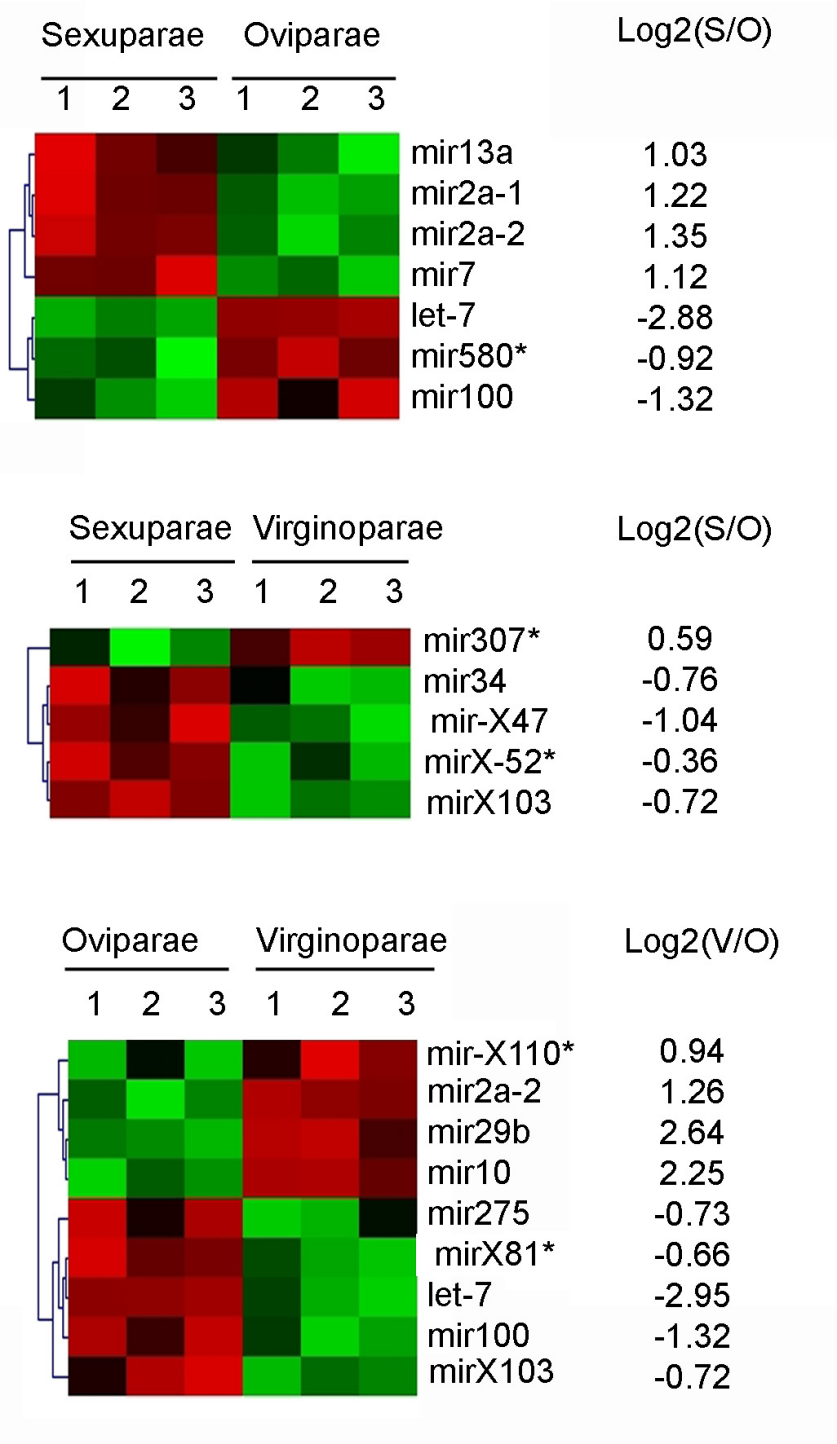
**Expression profiling of pea aphid miRNAs**. After microarray hybridization and statistical analyses, a set of pea aphid miRNAs differentially regulated in parthenogenetic females producing parthenogenetic females (Virginoparae, V), parthenogenetic females producing sexual individuals (Sexuparae, S) and sexual females (Oviparae, O) was identified. Three replicates are indicated (labelled 1 to 3) and profiles clustering are presented for miRNAs significantly regulated between morphs. Colour coding: red, up-regulated, green, down-regulated, black, not regulated). The log score for fold change is indicated.

The expression of the 149 pea aphid miRNAs identified in this work was followed in the different female morphs using a microarray. The array included one probe for each mature aphid miRNAs and its corresponding mir* and diverse controls (see methods). Each probe was repeated 10 times in the array. Small RNAs for hybridization were extracted from virginoparae, sexuparae and oviparae females, in three independent experiments. The large majority of the mature miRNAs hybridized to the chips; only four mature miRNAs (two conserved and two new miRNAs) and nine mir* gave no signal with any of the hybridizations. Most miRNAs and mir* (95%) showed no statistically significant differences in their expression between the morphs. However, 17 miRNAs (12 mature miRNAs and 5 mir*) showed significant differences in their steady-state levels between the morphs (Fig 3). These 17 miRNAs included 15 microRNAs and 2 microRNA candidates (Ap-mir-X103 and Ap-mir-X110): 12 conserved miRNAs and 5 new aphid miRNAs. Seven microRNAs were differentially expressed between sexual oviparae and sexuparae, and 9 were differentially expressed between sexual oviparae and virginoparae.

Interestingly, among them, Ap-let-7, Ap-mir2a-1 and Ap-mir100 showed differential expression between oviparae and the two other parthenogenetic morphs (virginoparae and sexuparae). Ap-let-7 and Ap-mir-100 were up-regulated in oviparae, and Ap-mir2a-1 was down-regulated in oviparae. Ap-let-7 and Ap-mir100 belong to the same cluster and follow similar expression patterns. Mir-2 is involved in apoptosis regulation during development [31] and let-7 and mir-100 have been reported to be involved in metamorphosis [32] and are up-regulated in response to ecdysone [32], a hormone involved in insect development. Three miRNAs (Ap-mir-34, Ap-mir-X47 and Ap-mir-X103) and two mir* (Ap-mir307* and Ap-mirX-52*) showed different expression between the two parthenogenetic morphs: sexuparae and virginoparae, that differ by the type of embryos they contain (sexual *vs *asexual), and by the conditions of rearing (short-day *vs *long-day). Four of these miRNAs were specific to the virginoparae/sexuparae comparison, while Ap-mir-X103 was also up-regulated in oviparae. These 5 miRNAs are particularly interesting candidates for the switch of reproduction mode from parthenogenesis to sexual reproduction. Three of these miRNAs are newly-identified aphid miRNAs and their function remains to be determined. Interestingly, mir-34 has been shown to be regulated in *D. melanogaster *by ecdysone as well as by juvenile hormones [32]. Juvenile hormones are known to be involved in aphids in the transduction of the photoperiodic signal from the brain to the ovaries during the switch of reproductive mode [33]. Thus, our study has identified a strong candidate (Ap-mir-34) as a putative regulator involved in reproductive polyphenism in aphids and opens new avenues for further functional analyses.

## Conclusions

This work has established a catalog of miRNA genes in the pea aphid that represents an essential base of knowledge-base for investigating the miRNA post-transcriptional regulation of key biological traits for an organism whose adaptation is shaped by phenotypic plasticity. Deciphering the gene regulation network between miRNAs and their mRNA targets in the pea aphid remains an objective that this study opens.

## Methods

### Biological material

The LSR1 clone [15] of the pea aphid *Acyrthosiphon pisum *was reared and maintained as clonal individuals (parthenogenesis) on the plant *Vicia fabae *at 18°C under a 16 h photoperiod for long-day condition (parthenogenesis). Sexual individuals and sexuparae were produced by rearing the pea aphid at 18°C under a 12 h photoperiod (short-day condition) as described in [34].

### Homology identification of *A. pisum *miRNAs

For homology prediction of *A. pisum *miRNAs, insect miRNAs (*D. melanogaster *and the 11 other *Drosophila *species, *A. mellifera, A. gambiae, B. mori, T. castaneum *and *Locusta migratoria*) were retrieved from miRBase release 14 [7] and blasted against the pea aphid genome. Putative miRNAs were selected as sequences identical or slightly identical (with 1 or 2 nt different) with the original homologous mature miRNA sequence retrieved from miRBase. A sliding window of 150 bases of genomic sequences surrounding each putative miRNAs were retrieved and its ability to fold into a potential miRNA hairpin precursor was investigated using the RNA fold program [35] and/or the MiRAlign program [36]). Secondary structure should have free energy change less than or equal to -18 kcal/mole.

To investigate the conservation of mature aphid miRNAs with other miRNAs listed in miRBase was analysed using the Similarity SEARCH program (SSEARCH) available on the miRBase website with default parameters. A minimum SSEARCH score of 135 was selected to be significant.

All miRNAs were posted to miRBase (Additional file 3). All *A. pisum *miRNA sequences have been mapped on the genome at AphidBase http://www.aphidbase.com.

### Small RNA sequencing

Total RNA was extracted from a mixed culture of parthenogenetic females of the LSR1 clone [15] of pea aphid using PureZOL RNA Isolation Reagent (Bio-Rad, Hayward CA). The RNA concentration and purity were determined photometrically by measuring absorbance at 260 nm and A260/A280 ratio using the NanoDrop ND-1000 spectrophotometer (Nanodrop Technologies). 40 μg of ethanol precipitated RNA was sent in duplicate to Illumina Inc. (Hayward CA) for size fractionation (<50 bp) and deep sequencing. Results of small RNA sequencing have been deposited in Gene Expression Omnibus (Additional file 4).

### Mapping of the short reads Illumina sequences on the pea aphid genome

Approximately 3 million total sequences were processed to remove linker sequences and quantify the resulting unique sequences. The 851,979 unique sequences (mean: 25.2 bp, median: 26 bp, max: 33 bp, min: 1 bp) were compared to the *A. pisum *genome using NCBI megablast (wod size: 12, no filter). For further analysis, we used an extremely selective process by conserving only exact match on the complete short read. Sequences that mapped to more than 5 different places on the genome were removed. It resulted in 305,055 alignments, covering only 205,500 (24%) of the unique reads, but 44% (1,333,398) of all reads.

### MiRDeep analysis

MiRDeep analysis was done according to the miRDeep protocol, and a data set of putative precursor sequences was obtained from the genome, based on the predicted mature miRNA alignments. The secondary structures of the putative precursors were predicted by RNAfold [35]. Short reads were aligned to their putative genomic precursor to create signatures of the precursors. Finally, we run miRDeep with the RNAfold stability test using those structures and signatures inputs.

### GR4500 analysis

The pea aphid genome was scanned to find miRNA-like hairpins. For that, we first used RNAfold [35] on 120 nucleotides windows with an overlap of 100 nucleotides, and selected in each window all hairpins longer than 63 bases with at least 70% arm base pairing. This led to approximately 2.5 million genomic hairpins. We then developed a set of features to discriminate between miRNA and non miRNA hairpins: folding energy, total and maximum internal loop size and symmetry, arms and terminal loop sizes; GC%, complexity score (in terms of repetitions of small motifs) and the score delivered by the Microprocessor SVM [37] which characterizes the presence of a Drosha recognition site. Using 38 pea aphid miRNAs already found by homology search as a learning set, we constructed a classifier using all these features. We first used a decision tree implemented in the R tree package to build a prototype classifier and then hand-tuned it to improve its selectivity. Additional file 5 lists the features and their corresponding cut-off thresholds. 4402 hairpins were selected and used for short reads alignment to create signatures of the precursors. Finally, as a last step, MiRDeep parametered with the sensitive option (-x) was used to extract mature sequences using those signatures and structures.

### MiRNAs profiling in three different morphs of the pea aphid

Based on the sequence of aphid mature miRNAs, we designed probes. The custom μparaflo™ microfluidic chip (LC Sciences Houston, USA) contained one probe for each mature aphid miRNA and its corresponding mir*. Each probe was repeated 10 times on the chip to ensure assay reproducibility. Multiple control probes were included on each chip for quality controls, sample labelling and assay conditions. Among the control probes, PUC2PM-20B and PUC2 MM-20B are the perfect match and single-based match detection probes, respectively, of a 20-mer RNA positive control sequence that is spiked into the RNA samples before labelling. The detection probes were made by *in situ *synthesis using PGR (photogenerated reagent) chemistry. The expression of miRNAs was analysed in three different adult morphs: parthenogenetic females reared under long day condition that produce parthenogenetic embryos, parthenogenetic females reared under short day condition that produce sexual embryos, and sexual females reared under short day condition that produce eggs. Total RNAs were extracted from 10 adult females 48 h after the final moult. Three independent samples were analysed for each reproductive morph. Total RNAs were extracted using miRNeasy purification kit (QIAGEN) according to manufacturer's instructions. Quality of RNAs was checked with the Bioanalyser (Agilent). The assay started from 5 μg total RNA sample, which was size fractionated using a YM-100 Microcon centrifugal filter (Millipore) and the small RNAs (< 300 bases) isolated were 3'-extended with a poly(A) tail using poly(A) polymerase. An oligonucleotide tag was then ligated to the poly(A) tail for later fluorescent dye staining; two different tags were used for the two RNA samples in dual-sample experiments. Hybridization was performed overnight on a μParaflo microfluidic chip using a micro-circulation pump (Atactic Technologies). On the microfluidic chip, each detection probe consisted of a chemically modified nucleotide coding segment complementary to target microRNA or other control RNAs and a spacer segment of polyethylene glycol to extend the coding segment away from the substrate. The hybridization melting temperatures were balanced by chemical modifications of the detection probes. Hybridization used 100 μL 6× SSPE buffer (0.90 M NaCl, 60 mM Na_2_HPO_4_, 6 mM EDTA, pH 6.8) containing 25% formamide at 34°C. After RNA hybridization, tag-conjugating Cy3 were circulated through the microfluidic chip for dye staining. Fluorescence images were collected using a laser scanner (GenePix 4000B, Molecular Device) and digitized using Array-Pro image analysis software (Media Cybernetics).

For each chip and each probe, the average signal value and its standard deviation were quantified. Data were analyzed by first subtracting the background, then integrating all the signals corresponding for the same probe for one given chip. A transcript to be listed as detectable must meets at least two conditions: signal intensity higher than 3× (background standard deviation) and spot CV < 0.5. CV. When repeating probes are present on an array, a transcript is listed as detectable only if the signals from at least 50% of the repeating probes are above detection level. Then normalization the signals from all arrays were performed using a LOWESS filter (Locally-weighted Regression). Results obtained in the different reproductive morphs were compared by comparing the ratio of the two sets of detected signals (log2) and p-values of the t-test were calculated. Differentially detected signals were those with p-value < 0.05. The results of microfluidic experiments have been deposited in Gene Expression Omnibus (Additional file 4).

## Authors' contributions

FL performed all the bioinformatic analyses and wrote a part of the manuscript. GR designed GR4500 algorithm together with DL and JN. TW was involved in the small RNA sequencing and data analysis, he took part in the writing of this manuscript. OE and KG discussed the data and were involved in the writing of this manuscript. NL was involved in the preparation of microRNAs samples for the microfluidic experiments. DT discussed the data and took part in the writing of the manuscript. SJP was in charge of the program, performed the biological experiments together with AM and wrote the manuscript. All the authors read and approved the final manuscript.

## Supplementary Material

Additional file 1 ***A. pisum *microRNAs**. list of predicted miRNAs from the pea aphid genome. The table presents the name, sequence of the mature *A. pisum *predicted miRNA, sequence of the hairpin precursor(s), reference of the genomic scaffold that includes the microRNA within the *A. pisum *genome, strand sense, method used for identification of the miRNA, clusterisation, genomic organisation (and identity of the protein coding gene, if the miRNA is predicted to be located within an exon) and conservation of the microRNA. The 11 mir* predicted as mature microRNA by miRDeep and/or GR4500 are indicated in this table but are shaded in grey. Click here for file

Additional file 2 Abundance of *A. pisum *miRNA candidates and their mir* within the Solexa reads. Abundance of *A. pisum *miRNA and miRNA candidates and their corresponding mir* within the the Solexa reads. *A. pisum *predicted microRNAs were designated as miRNAs if they have abundant reads (≥5) and/or their corresponding mir* were identified within the reads, otherwise they are designated as miRNA candidates. Click here for file

Additional file 3 **miRBase accession number**. miRBase accession number of *A. pisum *miRNAs. Click here for file

Additional file 4 **Sequencing of small RNas from *A. pisum *parthenogenetic colony**. The results of the sequencing of small RNAs from a parthenogenetic colony of *A. pisum *and of the results of the analysis by microfluidic of the expression of *A. pisum *microRNAs in various morphs have been deposited in Gene Expression Omnibus (Go). The GO accession numbers are provided Click here for file

Additional file 5 **GR4500 set up**. Set of features to discriminate using GR4500 between miRNA and non miRNA hairpins Click here for file
