# Supplementary material for: Bioinformatic prediction, deep sequencing of microRNAs and expression analysis during phenotypic plasticity in the pea aphid, Acyrthosiphon pisum
Source: BMC Genomics. 2010 May 5;11:281. doi: 10.1186/1471-2164-11-281 (PMC2880305; doi:10.1186/1471-2164-11-281)
Supplement: Additional file 5 — GR4500 set up. Set of features to discriminate using GR4500 between miRNA and non miRNA hairpins [file 1471-2164-11-281-S5.DOC]

Table 5 : Set of features to discriminate between miRNA and non miRNA hairpins, the threshold indicates the value used in the decision tree

| Features | Unit | Description | Threshold |
| --- | --- | --- | --- |
| AMFE | kcal/mol/100nt | minimum free energy /sequence length *100 | -55< AMFE <-34 |
| Unp | % | unpaired base percentage | Unp<0.27 |
| R1 | % | monomer repetition percentage | R1<0.17 |
| R2 | % | dimer repetition percentage | R2<0.09 |
| GC | % | GC percentage | 0.28<GC<0.50 |
| Asym_Max | nucleotide | Maximum asymmetry observed in a single loop, terminal loop region excluded | Asym_Max < 3 |
| Asym_Total | nucleotide | Total asymmetry observed over all loops, terminal loop region excluded | Asym_Total < 5 |
| Asym_Terminal | nucleotide | Maximum asymmetry observed in the terminal loop region | Asym_Terminal < 7 |
| Intern_Max | nucleotide | Maximum size of internal loop | Intern_Max < 9 |
| Intern_Total | nucleotide | Total size of internal loops | Intern_Total < 20 |
| Ll | nucleotide | left arm length | Ll > 25 |
| Lr | nucleotide | right arm length | Lr > 25 |
| Size | nucleotide | total sequence length | Size > 63 |
| SVM_drosha | synthetic score | score reported by Microprocessor SVM (Snorre A. Helvik et al. 2006) | SVM_drosha > -0.67 |
